# Taxonomic study of the genus *Neocarpia* Tsaur & Hsu, with descriptions of two new species from China (Hemiptera, Fulgoromorpha, Cixiidae)

**DOI:** 10.3897/zookeys.695.12809

**Published:** 2017-09-04

**Authors:** Yan Zhi, Lin Yang, Pei Zhang, Xiang-Sheng Chen

**Affiliations:** 1 Institute of Entomology, Guizhou University, Guiyang, Guizhou, 550025, P.R. China; 2 The Provincial Special Key Laboratory for Development and Utilization of Insect Resources of Guizhou, Guizhou University, Guiyang, Guizhou, 550025, P.R. China; 3 Laboratory Animal Center, Guizhou Medical University, Guiyang, Guizhou 550025, P.R. China; 4 Xingyi Normal University for Nationalities, Xingyi, Guizhou, 562400, P.R. China

**Keywords:** Female genitalia, Fulgoroidea, morphology, Oriental region, taxonomy

## Abstract

The cixiid planthoppers genus *Neocarpia* Tsaur & Hsu, 2003 is reviewed. Two new species, *N. acutata* Zhi & Chen, **sp. n.** and *N. reversa* Zhi & Chen, **sp. n.**, are described and illustrated from the southwest of China (Yunnan) to give the genus seven species in total. Female genitalia of four Chinese species are described and illustrated for the first time. A key to all known species of *Neocarpia* based on male genitalia, and a key to Chinese species (except for *N. maai*) based on female genitalia, are provided. The morphological characteristics of the posterior vagina, utilized to distinguish female species of *Neocarpia*, are also discussed.

## Introduction

Tsaur and Hsu (2003) established the cixiid planthopper genus *Neocarpia* with the type species *Neocarpia maai* Tsaur & Hsu, 2003 from China (Taiwan), and placed this genus in the tribe Pintaliini of the subfamily Cixiinae (Hemiptera: Fulgoromorpha: Cixiidae). Later, Emeljanov and Hayashi (2007) described *N. okinawana* from Japan and moved *Neocarpia* to the tribe Eucarpiini according to hind margin of the forewing without any convexity situated between the clavus apex and icu. So far, five species of *Neocarpia* are described, including three from China (Tsaur and Hsu 2003; Emeljanov and Hayashi 2007; Löcker et al. 2010; Zhang and Chen 2013).

Herein, two new species of *Neocarpia* are described and illustrated from Yunnan province, China. Female genitalia of four Chinese species are described and illustrated for the first time. The genus *Neocarpia* now contains seven species, including five from China. A key to species based on male genitalia, and to Chinese species (except for *N. maai*) based on female genitalia, are provided. The morphological characters of the posterior vagina are utilized to distinguish female species of *Neocarpia*.

## Materials and methods

The morphological terminology and measurements follow Tsaur et al. (1988) and Löcker et al. (2006) and the morphological terminology of female genitalia follows Bourgoin (1993). Body length was measured from apex of vertex to tip of forewing; vertex length was measured the median length of vertex (from apical transverse carina to tip of basal emargination). External morphology and drawings were done with the aid of a Leica MZ 12.5 stereomicroscope. Photographs of the types were taken with KEYENCE VHX-1000 system. Illustrations were scanned with CanoScan LiDE 200 and imported into Adobe Photoshop CS7 for labelling and plate composition. The dissected male and female genitalia are preserved in glycerine in small plastic tubes pinned together with the specimens.

The type specimens and other specimens examined are deposited in the Institute of Entomology, Guizhou University, Guiyang, Guizhou Province, China (**IEGU**).

## Taxonomy

### Neocarpia

Taxon classificationAnimaliaHemipteraCixiidae

Tsaur & Hsu, 2003

Neocarpia Tsaur & Hsu, 2003: 440; Löcker et al. 2010: 17; Zhang and Chen 2013: 42.

#### Type species.

*Neocarpia maai* Tsaur & Hsu, 2003, by original designation.

#### Emended diagnosis.

Head slightly narrower than pronotum in dorsal view. Vertex slightly widened to posterior emargination, broader than long and without subapical carina, lateral carinae moderately elevated. Frons with median carina; frontoclypeal suture generally angled or semicircular. Clypeus with well-developed median carina. Rostrum distinctly surpassing hind coxae. Pronotum short with intermediate carinae curved along posterior margins of eyes. Mesonotum tricarinate. Forewing in resting position steeply tectiform, widened towards apex, with rounded apical margin; Sc+R forming a common stem and M emerging separately from basal cell; MA trifid apically; position of fork Sc+R slightly basad or at the same level as fork CuA1+CuA2; first crossvein MP-CuA1 at least as long as MP from M fork to this crossvein, crossvein MP-CuA1 almost at same level as crossvein r-m, subapical cell MP with upper margin (vein MP) fine concave, no crossvein between CuA1 and CuA2. Apical cells 10. Hind tibia lacking lateral spines.

Male genitalia. Pygofer symmetrical and prolonged with symmetrical lateral lobes in lateral view. Medioventral process thumb-like in lateral view. Anal segment tubular, short and stout. Genital styles relative small and symmetrical. Aedeagus slender and flagellum of aedeagus with spinose processes.

Female genitalia. Ovipositor elongate, orthopteroid and slightly curved upwards; anal segment square or rectangular in dorsal view; 9^th^ tergite without wax plate. Gonapophysis VIII slightly sclerotised, blade-like posteriorly. Gonapophysis IX single, blunt and strongly sclerotised, between middle tooth and apex with a row of denticles. Gonoplac slightly sclerotised, with many spinules on ventral edge in inner lateral view. Posterior vagina with sclerites.

#### Remarks.

This genus may be easily distinguished from other genera of Eucarpiini by the following features: frontoclypeal suture generally angled or semicircular; rostrum distinctly surpassing hind coxae; forewing with ten apical cells, Sc+R forking slightly basad or at same level as fork CuA1+CuA2, first crossvein MP-CuA1 as long as or longer than vein MP from M fork to this veinlet, subapical cell MP with upper margin (vein MP) fine concave, no transverse vein between CuA1 and CuA2, position of first crossvein MP-CuA1 almost at same level as first crossvein r-m (Zhang and Chen 2013).

#### Distribution.

China, Japan, Australia.

### Checklist and distributions of species of *Neocarpia* Tsaur & Hsu

*N. acutata* Zhi & Chen, **sp. n.**; China (Yunnan).

*N. bidentata* Zhang & Chen, 2013; China (Guizhou).

*N. hamata* Zhang & Chen, 2013; China (Guizhou, Hubei).

*N. maai* Tsaur & Hsu, 2003; China (Taiwan).

*N. okinawana* Emeljanov & Hayashi, 2007; Japan (Ryukyus).

*N. reversa* Zhi & Chen, **sp. n.**; China (Yunnan).

*N. rhizophorae* Löcker, 2010; Australia (Queensland).

### Key to species (males) of *Neocarpia* (revised from Zhang and Chen 2013)

**Table:** 

1	Ventral margin of periandrium without spinose process	**2**
–	Ventral margin of periandrium with one or two spinose process(es)	**3**
2	Dorsal margin of periandrium with one process; flagellum with two processes near apex and without process at base (Emeljanov and Hayashi 2007: Figs 23–24)	***N. okinawana***
–	Dorsal margin of periandrium without process; flagellum with one process near apex and a long process near base (Figs 54–55)	***N. reversa* sp. n.**
3	Ventral margin of periandrium with one small triangular process at basal 1/3	**4**
–	Ventral margin of periandrium without triangular process at base, while with one or two process(es) near or at apex	**5**
4	Left side of periandrium with a process near apex, dorsal margin with a shovel-shaped process, right side without process in the middle, base of process near apex of flagellum with two denticulations (Zhang and Chen 2013: Figs 10–11); forewing without stripe	***N. bidentata***
–	Left side of periandrium without process, dorsal margin without process, right side with a short acute process in the middle, base of process near apex of flagellum without denticulation (Figs 12–13); forewing with yellow stripes along the Y-veins	***N. acutata* sp. n.**
5	Flagellum with a prominent long process in the middle (Löcker et al. 2010: Fig. 17A)	***N. rhizophorae***
–	Flagellum without process in the middle	**6**
6	Dorsal margin of periandrium with a hook-shaped process, ventral margin of periandrium with one process, flagellum with smooth apical margin (Zhang and Chen 2013: Figs 23–24)	***N. hamata***
–	Dorsal margin of periandrium without process, ventral margin of periandrium with two processes, flagellum with sinuate apical margin (Tsaur and Hsu 2003: Fig. 6D)	***N. maai***

### Key to Chinese species (females) of *Neocarpia* (except for *N. maai*)

**Table:** 

1	Posterior vagina without long longitudinal sclerite (Figs 41–42)	***N. hamata***
–	Posterior vagina with a long longitudinal sclerite	**2**
2	Long longitudinal sclerite on right side ventrally (Figs 65–66)	***N. reversa* sp. n.**
–	Long longitudinal sclerite on left side ventrally	**3**
3	Posterior vagina elongate, left side with two longitudinal sclerites; each side with a small sclerite near terminal in ventral view (Figs 23–24)	***N. acutata* sp. n.**
–	Posterior vagina relatively short, left side with one longitudinal sclerite; posterior vagina with a wide sclerite medially and a small longitudinal sclerite on the left side near terminal in ventral view (Figs 32–33)	***N. bidentata***

### Neocarpia acutata

Taxon classificationAnimaliaHemipteraCixiidae

Zhi & Chen sp. n.

http://zoobank.org/2AFE0126-5893-40A6-9044-E19D321F762E

Figs 1–4 , 5–15 , 16–24

#### Type material.

Holotype: ♂, **China**: Yunnan, Jinping County, Fenshuiling (22°86'N, 103°22'E), 8 June 2013, Liang-Jing Yang; paratypes: 1♂, 3♀♀, same data as holotype, Liang-Jing Yang and Ying-Jian Wang; 1♀, China: Yunnan, Pingbian County, Daweishan (22°81'N, 103°79'E), 5 June 2013, Liang-Jing Yang.

#### Description.

Body length: male 4.8–5.0 mm (*N* = 2), female 5.1–5.3 mm (*N* = 4); forewing length: male 4.5–4.8 mm (*N* = 2), female 4.8–5.0 mm (*N* = 4).

**Figures 1–4.**
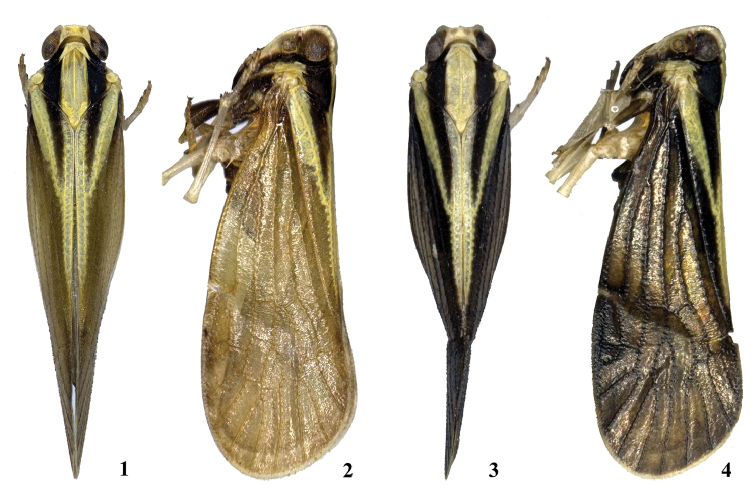
*Neocarpia acutata* sp. n. **1** Male, dorsal view **2** Male, lateral view **3** Female, dorsal view **4** Female, lateral view.

**Figures 5–15.**
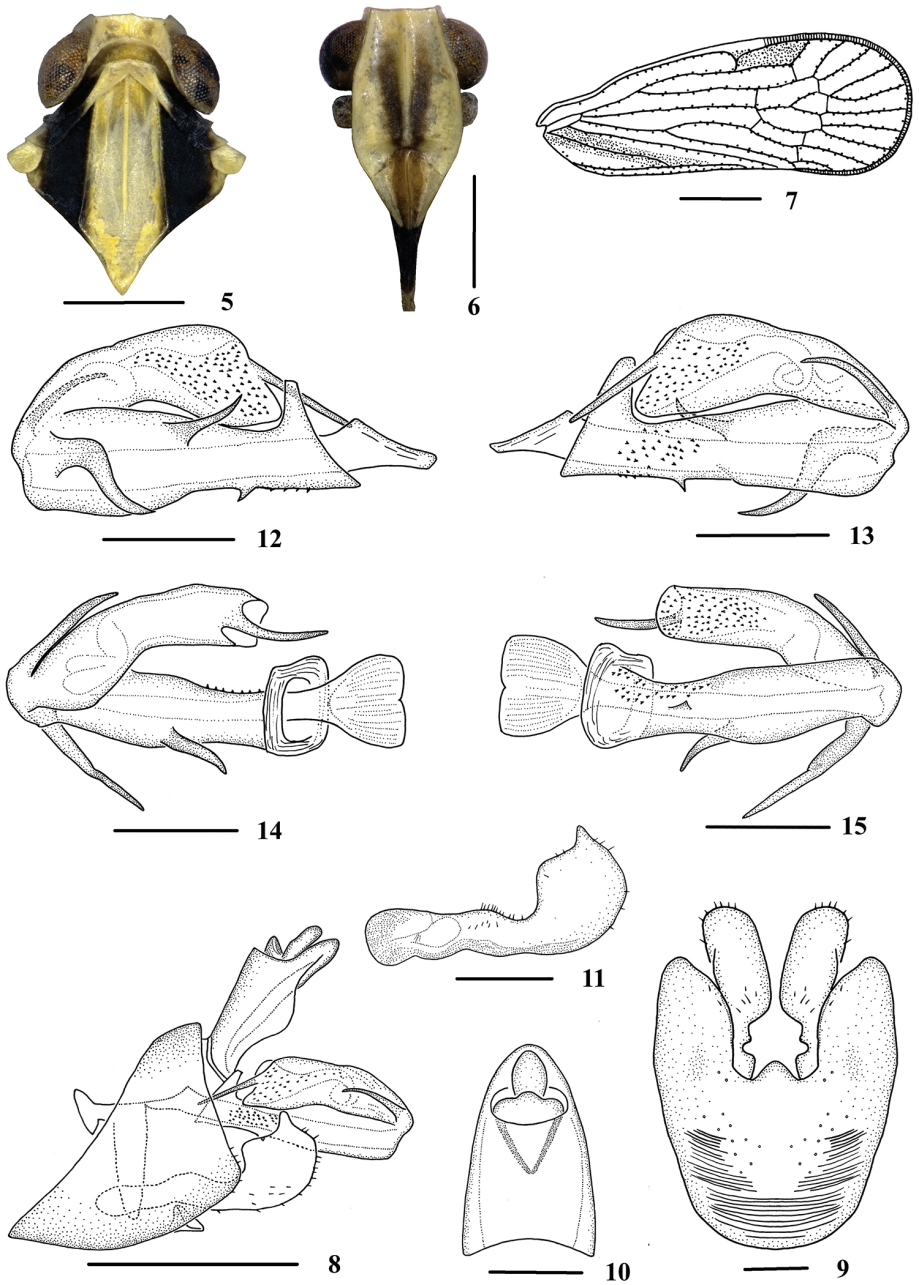
*Neocarpia acutata* sp. n., male **5** Head and thorax, dorsal view **6** Face, ventral view **7** Forewing **8** Genitalia, lateral view **9** Pygofer and genital styles, ventral view **10** Anal segment, dorsal view **11** Genital styles, inner lateral view **12** Aedeagus, right side **13** Aedeagus, left side **14** Aedeagus, dorsal view **15** Aedeagus, ventral view. Scale bars: 0.5 mm (**5–6, 8**); 1.0 mm (**7**); 0.2 mm (**9–15**).

*Coloration*. General color brown (Figs 1–6) (blackish brown in female). Eyes brown, ocelli pale yellow. Vertex generally yellow, carinae brown to dark brown (except median carina milky). Face generally yellow, discal area brown to dark brown. Subapical segment of rostrum blackish brown, apical segment brown with dark brownish apex. Pronotum with discal areas and mesonotum with area between lateral carinae yellow, lateral areas brownish black. Forewing semihyaline, brown throughout; yellow stripes along the Y-veins, the triangle area between the Y-veins brownish black. Hind tibiae pale yellow. Ventral abdomen blackish brown.

*Head and thorax*. Vertex (Figs 1, 3, 5) broad, 3.0 times wider than long; anterior margin slightly produced, posterior margin convexly recessed. Frons widest slightly below the level of antennae, 1.4 times as long as wide; frontoclypeal suture nearly concave into an arch; middle carina complete; lateral carinae distinct and elevated. Pronotum (Figs 1, 3, 5) 3.4 times longer than vertex; median carina indistinct, posterior margin nearly at right angle. Mesonotum 1.6 times longer than pronotum and vertex combined. Forewing (Figs 2, 4, 7) amply exceeding the tip of abdomen, 2.6 times longer than wide, with six subapical cells; fork Sc+RP slightly basad of fork CuA1+CuA2, first crossvein r-m slightly basad of fork MA+MP; RP and MP bifid separately; fork MA1+MA2 basad of fork MP1+MP2. Hind tibia with six apical spines; chaetotaxy of hind tarsi: 7/8.

*Male genitalia*. Pygofer (Figs 8, 9), dorsal margin shallowly concave and U-shaped ventrally, widened towards apex; in lateral view, lateral lobes triangularly extended caudally. Anal segment (Figs 8, 10), dorsal margin almost straight, ventral margin convex in lateral view, apical margin convex and 1.6 times longer than wide in dorsal view; anal style strap-shaped, not beyond anal segment. Apical margin of genital styles (Figs 8, 11) with a small blunt process, dorsal margin bending inwards in the middle. Aedeagus (Figs 12–15) with five spinose processes. Right side of periandrium with a long and broad process, strongly curving near apex directed ventrocephally and a short acute process curved in the middle directed dorsocephally; ventral margin with a small triangular process at basal 1/3, directed ventrocaudally; flagellum moderately sclerotised, generally curved on left side; left side with a short process basally, curved and directed cephalad, and a straight process at apex directed ventrocephally.

**Figures 16–24.**
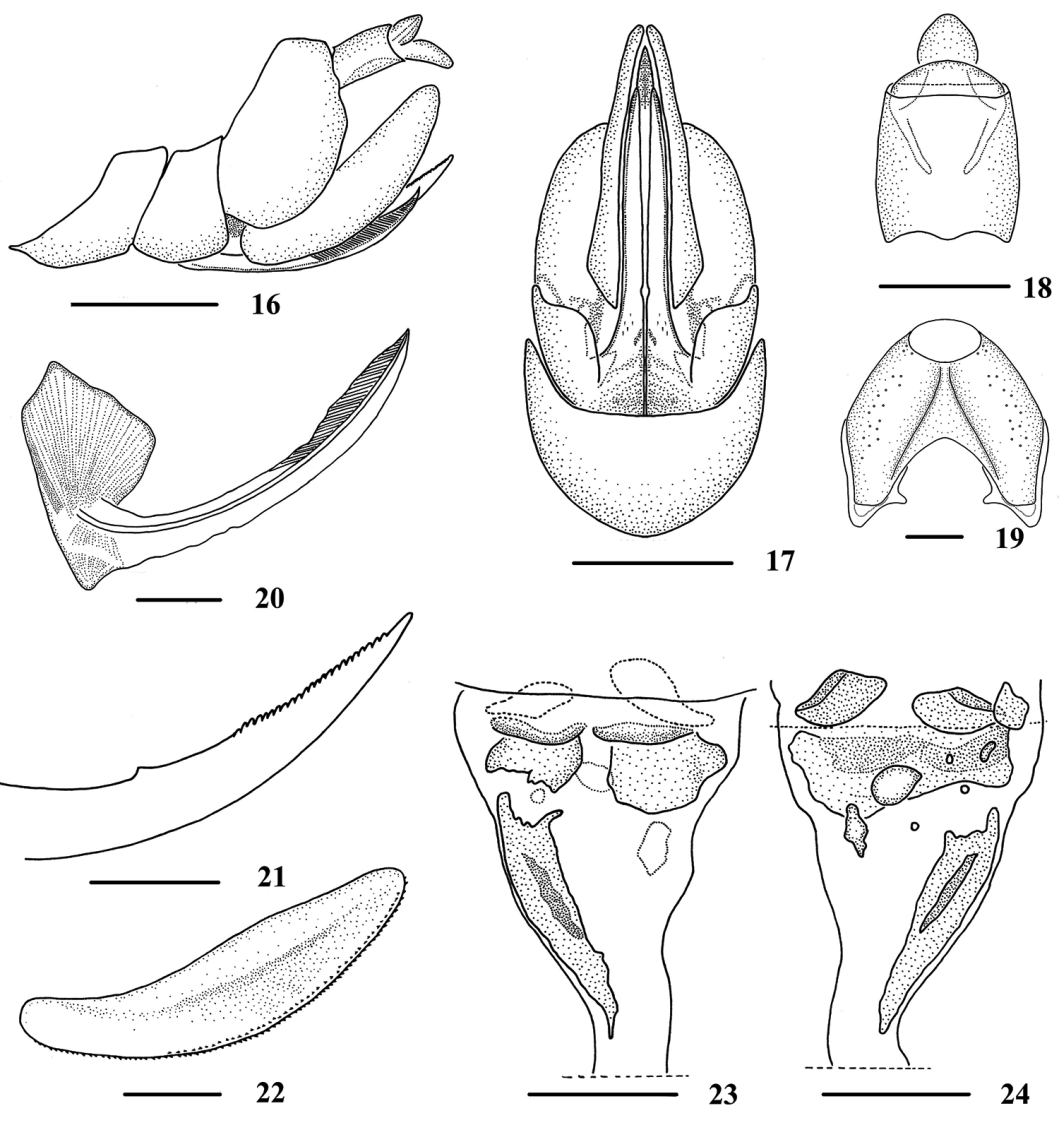
*Neocarpia acutata* sp. n., female **16** Genitalia, lateral view **17** Genitalia, ventral view **18** Anal segment, dorsal view **19** Pygofer, caudal view **20** Gonapophysis VIII and gonocoxa VIII, dorsal view **21** Gonapophysis IX, lateral view **22** Gonoplac, inner lateral view **23** Posterior vagina, ventral view **24** Posterior vagina, dorsal view. Scale bars: 0.5 mm (**16–17**); 0.2 mm (**18–24**).

*Female genitalia*. Pygofer (Figs 16–17, 19) moderately sclerotised, with length almost equal to width in caudal view. Anal tube (Figs 16, 18) short, length longer than wide in dorsal view, ventral margin straight in lateral view; anal styles relatively short and small, apical margin semicircular in dorsal view. Gonapophysis VIII (first valvula) (Fig. 20) elongate, and slightly curved upwards, 2/5 of its inner margin sinuate basally. Gonapophysis IX (second valvula) (Fig. 21), distance ratio between middle tooth to apex and length of denticulate portion is 1.72. Gonoplac (third valvula) (Fig. 22) rod-like, 4.2 times longer than wide, with width of spiculated area less than its 1/10. Posterior vagina (Figs 23–24) elongate, at terminal each lateral side with a sclerite respectively in ventral view; with a large transverse sclerite and several small sclerites in dorsal view; a long longitudinal sclerite in ventral view and a much shorter one in dorsal view on left side basally.

#### Distribution.

China (Yunnan).

#### Etymology.

The specific name is derived from the Latin word “*acutata*”, referring to the right side of periandrium bearing an acute process in the middle near dorsal margin.

#### Remarks.

Male genitalia of *N. acutata* sp. n. is similar to *N. bidentata* Zhang & Chen, 2013, but differs in: (1) right side of periandrium near dorsal margin with a short acute process in the middle curved and directed dorsocephally (in *N. bidentata*, right side without process in the same position); (2) right side of periandrium with a long and broad process strongly curved near apex directed ventrocephally (process on right side of periandrium near apex straight and directed dorsocephally in *N. bidentata*); (3) left side of flagellum with a process basally (in *N. bidentata*, without process in the same position).

Female genitalia of *N. acutata* sp. n. is similar to *N. bidentata* Zhang & Chen, 2013, but differs in: (1) posterior vagina elongate, left side with two longitudinal sclerites (in *N. bidentata*, posterior vagina relatively short, left side with one longitudinal sclerite); (2) each side of posterior vagina with a small sclerite near terminal in ventral view (in *N. bidentata*, posterior vagina with a wide sclerite medially and a small longitudinal sclerite on the left side near terminal in ventral view).

### Neocarpia bidentata

Taxon classificationAnimaliaHemipteraCixiidae

Zhang & Chen, 2013

Figs 25–33

Neocarpia bidentata Zhang & Chen, 2013: 43: figs 1–13; 47: 27–29.

#### Material examined.

1♂, **China**: Guizhou, Xishui County, Linjiang, 1 June 2006, Xiang-Sheng Chen (Holotype); 3♀♀, same data as holotype (Paratypes); 1♂, China: Guizhou, Wangmo County, Dayi, 24 September 1997, Xiang-Sheng Chen (Paratype).

#### Supplementary description.

**Figures 25–33.**
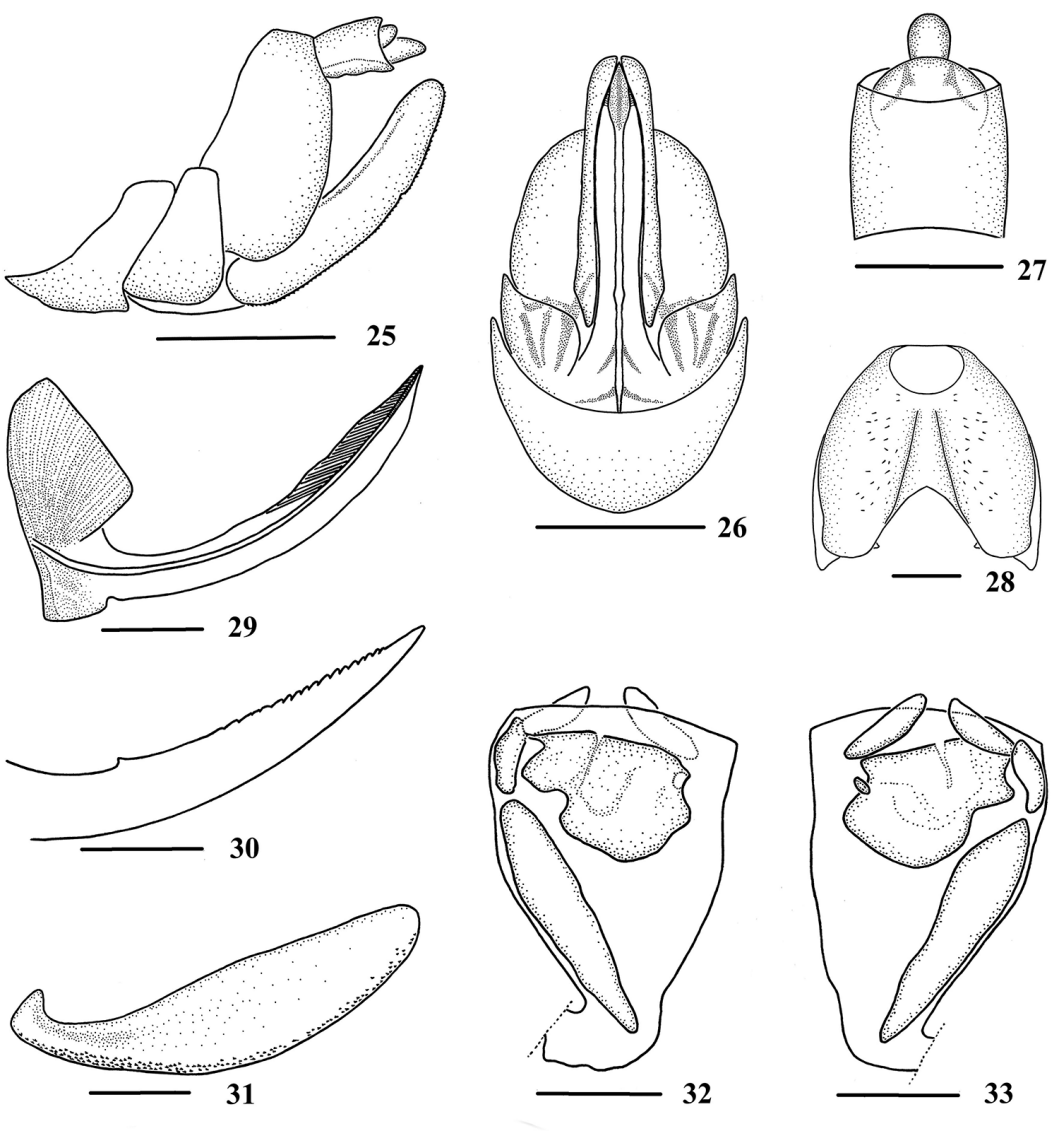
*Neocarpia bidentata* Zhang & Chen, 2013, female **25** Genitalia, lateral view **26** Genitalia, ventral view **27** Anal segment, dorsal view **28** Pygofer, caudal view **29** Gonapophysis VIII and gonocoxa VIII, dorsal view **30** Gonapophysis IX, lateral view **31** Gonoplac, inner lateral view **32** Posterior vagina, ventral view **33** Posterior vagina, dorsal view. Scale bars: 0.5 mm (**25–26**); 0.2 mm (**27–33**).

*Female genitalia*. Pygofer (Figs 25–26, 28) moderately sclerotised, slightly shorter than wide in caudal view. Anal tube (Figs 25, 27) short, slightly longer than wide in dorsal view, ventral margin sinuate in lateral view; anal styles relatively short and small, strap-like. Inner margin of gonapophysis VIII (Fig. 29) concave near base. Gonapophysis IX and gonoplac (Figs 30–31) same as in *N. acutata*, while the width of spiculated area approximately 1/10 of gonoplac, length of gonoplac 4.3 times of its width. Posterior vagina (Figs 32–33) stubby, with a wide sclerite medially and a small longitudinal sclerite on left side near terminal, left side with a long longitudinal sclerite.

#### Distribution.

China (Guizhou).

#### Remarks.

Diagnosis of female see *Neocarpia acutata* Zhi & Chen, sp. n.

#### Note.

The female genitalia of this species is described and illustrated for the first time.

### Neocarpia hamata

Taxon classificationAnimaliaHemipteraCixiidae

Zhang & Chen, 2013

Figs 34–42

Neocarpia hamata Zhang & Chen, 2013: 45: figs 14–26; 47: 30–32.

#### Material examined.

1♂, **China**: Guizhou, Yanhe County, Daheba, 5–12 June 2007, Pei Zhang (Holotype); 1♀, same data as holotype (Paratype); 3♀♀, China: Guizhou, Yanhe County, Lijiaba, 5–12 June 2007, Pei Zhang (Paratypes); 19♂♂, 16♀♀, China: Hubei, Luotian County, Qingtaiguan, (31°16'N, 115°69'E), 29 June–3 July 2014, Zhi-Min Chang, Zheng-Xiang Zhou and Mei-Na Guo.

#### Supplementary description.

**Figures 34–42.**
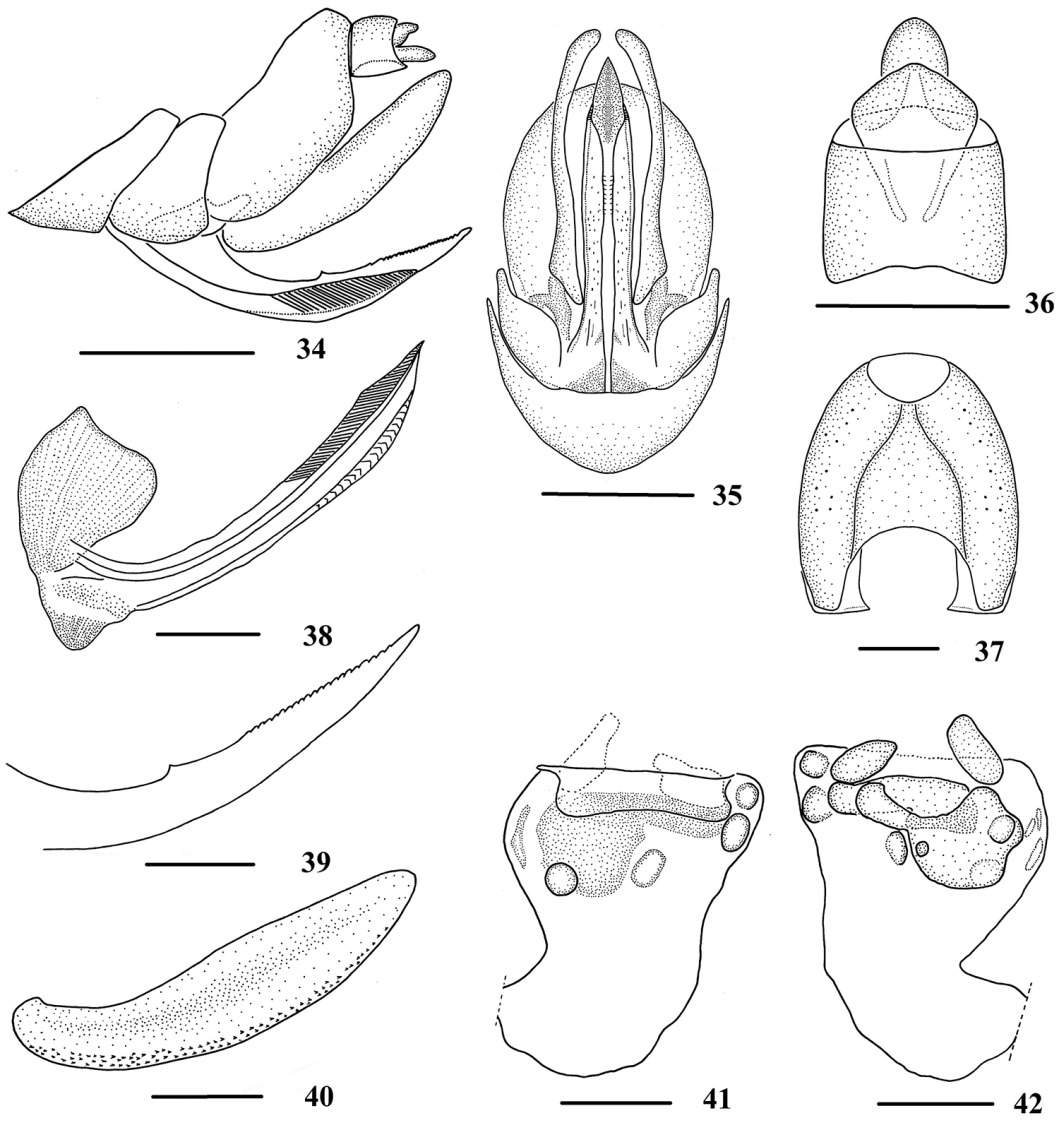
*Neocarpia hamata* Zhang & Chen, 2013, female **34** Genitalia, lateral view **35** Genitalia, ventral view **36** Anal segment, dorsal view **37** Pygofer, caudal view **38** Gonapophysis VIII and gonocoxa VIII, dorsal view **39** Gonapophysis IX, lateral view **40** Gonoplac, inner lateral view **41** Posterior vagina, ventral view **42** Posterior vagina, dorsal view. Scale bars: 0.5 mm (**34–35**); 0.2 mm (**36–42**).

*Female genitalia*. Pygofer (Figs 34–35, 37) moderately sclerotised, 1.2 times longer than wide in caudal view. Anal tube (Figs 34, 36) short, shorter than wide in dorsal view, ventral margin slightly convex in lateral view; anal styles relatively short and small, strap-like. Gonapophysis VIII and IX and gonoplac (Figs 38–40) same as in *N. acutata*, while width of spiculated area approximately 1/8 of gonoplac, length of gonoplac 4.6 times of its width. Posterior vagina (Figs 41–42) stubby, with a long transverse sclerite near terminal, an irregular sclerite (left edge large and right edge small) and several circular or oval ones in dorsal view, without sclerite near base.

#### Distribution.

China (Guizhou, Hubei).

#### Remarks.

Female of *N. hamata* is similar to *N. acutata* sp. n., but differs in: (1) posterior vagina without sclerite near base (posterior vagina with two longitudinal sclerites near base in *N. acutata*); (2) anal tube shorter than wide in dorsal view (in *N. acutata*, anal tube longer than wide in dorsal view).

#### Note.

The female genitalia of this species is described and illustrated for the first time.

### Neocarpia maai

Taxon classificationAnimaliaHemipteraCixiidae

Tsaur & Hsu, 2003

Neocarpia maai Tsaur & Hsu, 2003: 441: fig. 6A–H.

#### Distribution.

China (Taiwan).

**Remarks.** Based on the description and the figures by Tsaur and Hsu (2003), this species can be distinguished from other species of the genus by the following characters: ventral margin of periandrium of aedeagus with 2 processes near apex; one process implanted on right side of periandrium near apex; flagellum with sinuate apical margin, a small awl-shaped production protruding on left side near apex.

### Neocarpia okinawana

Taxon classificationAnimaliaHemipteraCixiidae

Emeljanov & Hayashi, 2007

Neocarpia okinawana Emeljanov & Hayashi, 2007: 128: figs 4–5; 135: figs 21–24.

#### Distribution.

Japan (Ryukyus).

#### Remarks.

Based on the description and the figures by Emeljanov and Hayashi (2007), this species can be distinguished from other species of the genus by the following characters: periandrium bearing two processes on left side and one on right side near apex; dorsal margin of periandrium with one process, directed caudally; flagellum with two processes near apex.

### Neocarpia reversa

Taxon classificationAnimaliaHemipteraCixiidae

Zhi & Chen sp. n.

http://zoobank.org/B4C95EA6-2EE4-446B-9BF3-72E4339216A3

Figs 43–46 , 47–57 , 58–66

#### Type material.

Holotype: ♂, **China**: Yunnan, Xichou County, Fadou (23°38'N, 104°78'E), 28 June 2013, Ying-Jian Wang; paratypes: 11♂♂, 29♀♀, same data as holotype, Ying-Jian Wang and Qiang Luo.

#### Description.

Body length: male 5.8–6.3 mm (*N* = 7), female 6.3–6.6 mm (*N* = 20); forewing length: male 5.0–5.3 mm (*N* = 7), female 5.1–5.8 mm (*N* = 20).

**Figures 43–46.**
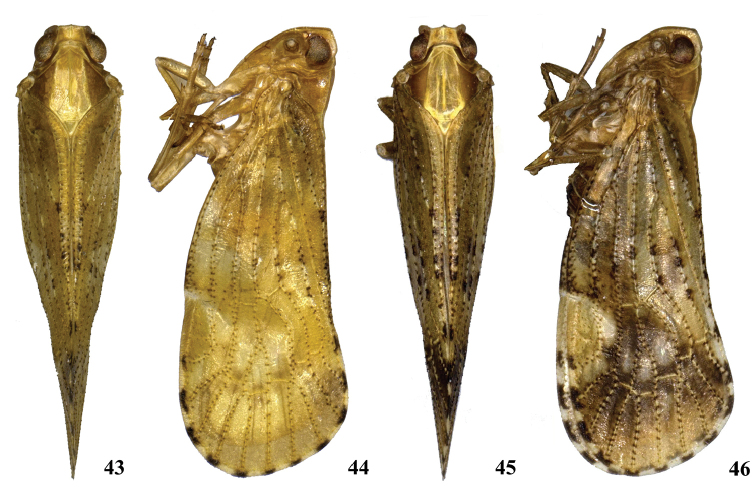
*Neocarpia reversa* sp. n. **43** Male, dorsal view **44** Male, lateral view **45** Female, dorsal view **46** Female, lateral view.

**Figures 47–57.**
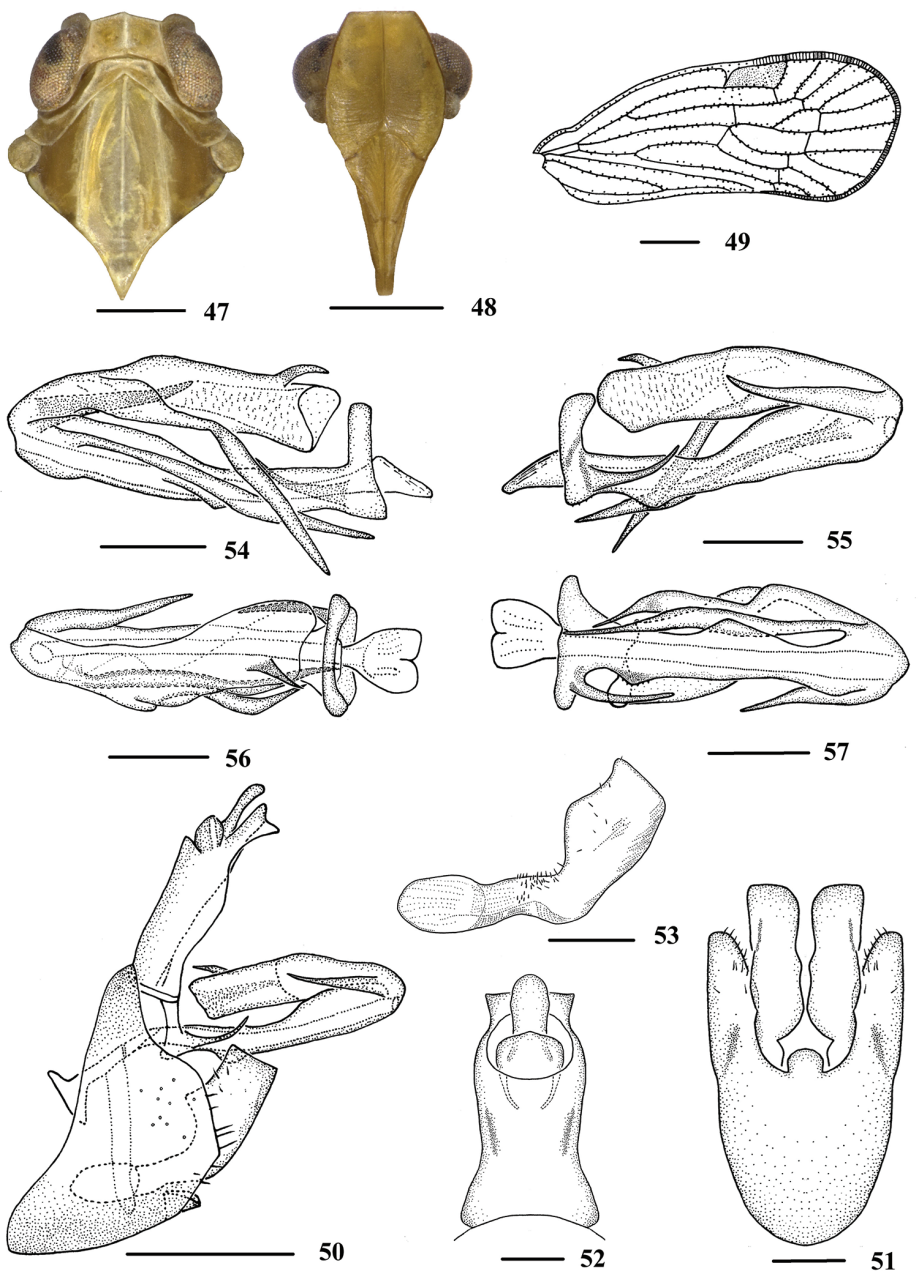
*Neocarpia reversa* sp. n., male **47** Head and thorax, dorsal view **48** Face, ventral view **49** Forewing **50** Genitalia, lateral view **51** Pygofer and genital styles, ventral view **52** Anal segment, dorsal view **53** Genital styles, inner lateral view **54** Aedeagus, right side **55** Aedeagus, left side **56** Aedeagus, dorsal view **57** Aedeagus, ventral view. Scale bars: 0.5 mm (**47–48, 50**); 1.0 mm (**49**); 0.2 mm (**51–57**).

*Coloration.* General color yellowish brown (Figs 43–48) (brown in female). Eyes brown, ocelli yellow. Vertex generally yellowish brown, carinae brown to dark brown (except median carina milky). Face generally yellow, carinae brown to dark brown; rostrum yellowish brown with dark brownish apex. Pronotum and mesonotum with areas between lateral carinae yellow, lateral areas brown. Forewing semihyaline, alternately yellowish brown and pale yellowish brown, with black spots on end of longitudinal veins. Hind tibiae yellowish brown. Ventral abdomen yellowish brown.

*Head and thorax*. Vertex (Figs 43, 45, 47) broad, 2.0 times wider than long; anterior margin slightly projected, posterior margin convexly recessed. Frons same as *N. acutata*. Pronotum (Figs 43, 45, 47) 2.1 times longer than vertex; median carina indistinct, posterior margin rather right-angled. Mesonotum 1.7 times longer than pronotum and vertex combined. Forewing (Figs 44, 46, 49) amply exceeding tip of abdomen, 2.4 times longer than wide, other veins same as *N. acutata*. Hind tibia with 6 apical spines; chaetotaxy of hind tarsi: 5/7.

*Male genitalia*. Pygofer (Figs 50, 51), same as *N. acutata*. Anal segment (Figs 50, 52), in lateral view, dorsal margin nearly straight, ventral margin slightly convex, with a horn-like process extending to apex ventrally; in dorsal view 1.8 times longer than wide; anal style strap-like, beyond anal segment. Apical margin of genital styles (Figs 50, 53) with a small blunt process, dorsal margin bending inwards in the middle. Aedeagus (Figs 54–57) with five spinose processes. Right side of periandrium with a very long process near apex directed ventrocephally. Left side of periandrium with a short reversed process at base directed dorsocaudally, and a medium sized process near apex directed dorsocephally. Flagellum moderately sclerotised. Right side with a long process near base directed ventrocephallly. Apex near dorsal margin with a short process, curved towards cephalad.

**Figures 58–66.**
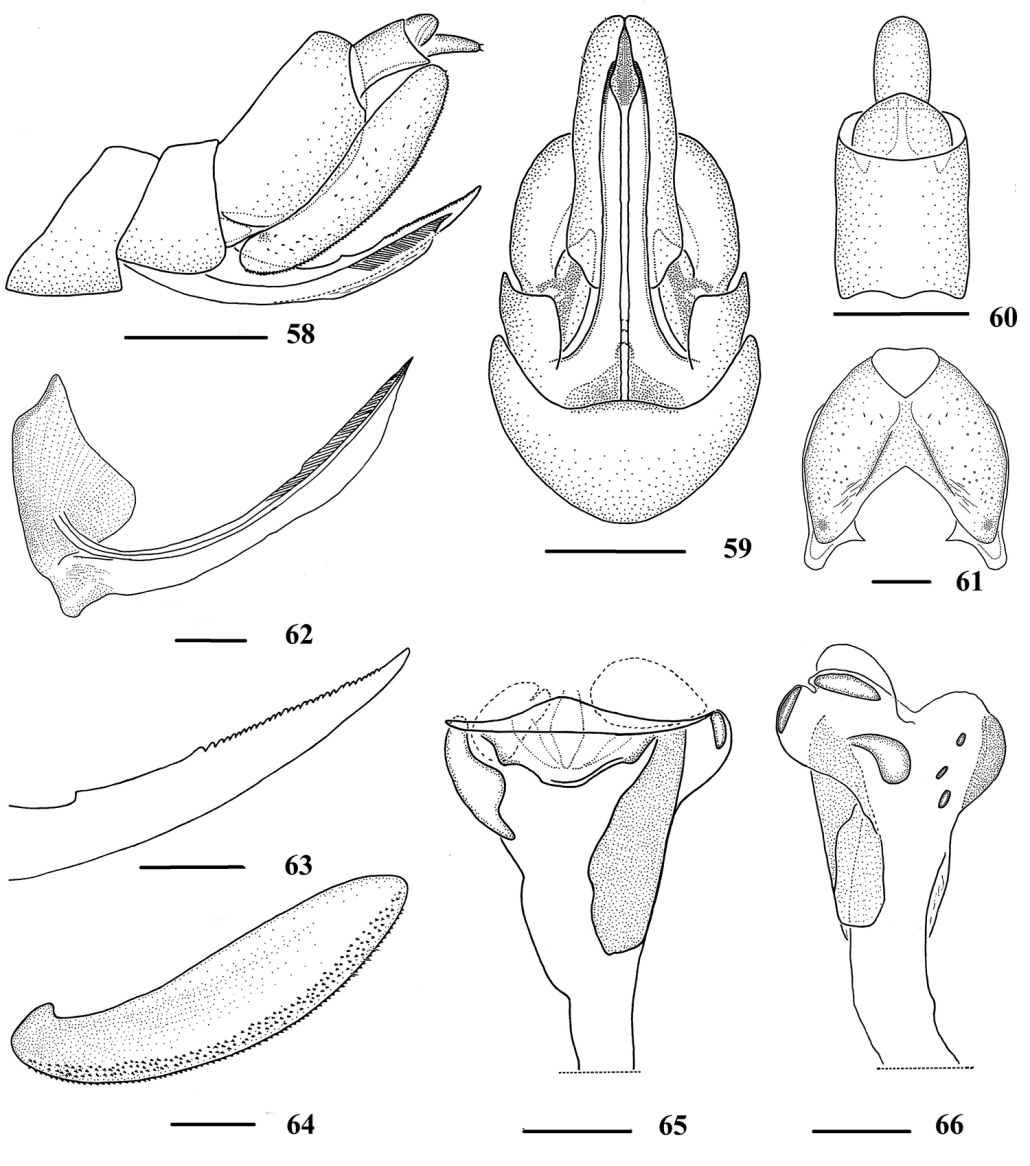
*Neocarpia reversa* sp. n., female **58** Genitalia, lateral view **59** Genitalia, ventral view **60** Anal segment, dorsal view **61** Pygofer, caudal view **62** Gonapophysis VIII and gonocoxa VIII, dorsal view **63** Gonapophysis IX, lateral view **64** Gonoplac, inner lateral view **65** Posterior vagina, ventral view **66** Posterior vagina, dorsal view. Scale bars: 0.5 mm (**58–59**); 0.2 mm (**60–66**).

*Female genitalia*. Pygofer (Figs 58–59, 61) same as in *N. acutata*. Anal tube (Figs 58, 60) short, 1.2 times longer than wide in dorsal view, ventral margin slightly concave in lateral view; anal styles relatively short and small, finger-shaped. Gonapophysis VIII (Fig. 62) slightly concave basally. Gonapophysis IX and gonoplac (Figs 63–64) same as in *N. acutata*, while length of gonoplac 4.3 times of its width, and width of spiculated area approximately 1/5 of gonoplac. Posterior vagina (Figs 65–66) elongate, right side with a long longitudinal sclerite in ventral view and a shorter one in dorsal view, forming a cylindrical structure, left side with a moderately long sclerite in ventral view, hat-shaped. In dorsal view, middle area of posterior vagina with a drop-like sclerite, right side with two small oblong sclerites near terminal.

#### Distribution.

China (Yunnan).

#### Etymology.

The specific name is derived from the Latin word “*reversa*”, referring to the left side of the periandrium with a short reversed process basally.

#### Remarks.

Male genitalia of *N. reversa* sp. n. is similar to *N. maai* Tsaur & Hsu, 2003, but differs in: (1) left side of periandrium with a short reversed process basally (*N. maai* without process in same position); (2) two processes on both lateral sides of periandrium near apex (three processes on periandrium near apex, two on ventral margin and one on right side in *N. maai*); (3) flagellum with smooth apical margin (sinuate in *N. maai*).

Female genitalia of *N. reversa* is similar to *N. bidentata* Zhang & Chen, 2013, but differs in: posterior vagina with a long longitudinal sclerite on left side (posterior vagina with a long longitudinal sclerite on right side in *N. bidentata*).

### Neocarpia rhizophorae

Taxon classificationAnimaliaHemipteraCixiidae

Löcker in Löcker, Fletcher & Gurr, 2010

Neocarpia rhizophorae Löcker, in Löcker, Fletcher & Gurr, 2010: 18: fig. 7A–D; 28: fig. 17A–E.

#### Distribution.

Australia (Queensland).

#### Remarks.

Based on the description and the figures by Löcker et al. (2010), this species can be distinguished from other species of the genus by the following characters: right side of periandrium with a process near apex and ventral margin of periandrium with one small triangular process at apical 1/3; flagellum with a prominent long process in the middle.

## Discussion

The taxonomic characters of cixiid male genitalia have been sufficiently studied, whereas the descriptions of cixiid female genitalia are quite rare. Although some characters of the female external genitalia like ovipositor, anal segment, anal style and wax plate have been described by several researchers in history, such as: *Cixius* Latreille (Remane and Asche 1979), *Hyalesthes* Signoret (Sforza and Bourgoin 1998), *Trirhacus* and related taxa (Holzinger 2002) and *Oteana* Hoch (Hoch 2006), these morphological characters are reported only reliable in taxonomic identifications on genus level or higher category, applying them in species identifications is often impracticable (Holzinger et al. 2002; Löcker et al. 2006). Nonetheless, using the characters of female inner genitalia structures, especially those such as the sclerites on the walls of the posterior vagina may provide a practical way for the species level identifications of the female cixiids (Bourgoin 1993; Holzinger et al. 2002; Orosz 2013).

Tsaur and Hsu described and illustrated the female pygofer and the anal segment of *Neocarpia maai* (Tsaur and Hsu 2003). Löcker et al. (2010) reported the morphological characters of the ovipositor, the 8^th^ abdominal sternite, the anal segment and the anal style of *N. rhizophorae*. However, these characters are not effective when used to distinguish among species of *Neocarpia*. Combined with the type specimens of *Neocarpia*, we found that the characteristics of posterior vaginal walls (Figs 23–24, 32–33, 41–42, 65–66) can be considered as key diagnostic features for female species identification and might provide evidence for the species diagnosis for other *Neocarpia* and Cixiidae. The variety of sclerites in numbers, sizes, and shapes in the walls of the female posterior vagina may be of high potential value in species identification in Cixiidae. In future study, we suggest that the morphological characters of the posterior vagina should be given more attention and their effectiveness in species identifications can be better evaluated and explored through more descriptions and illustrations of this structure.

## Supplementary Material

XML Treatment for Neocarpia

XML Treatment for Neocarpia acutata

XML Treatment for Neocarpia bidentata

XML Treatment for Neocarpia hamata

XML Treatment for Neocarpia maai

XML Treatment for Neocarpia okinawana

XML Treatment for Neocarpia reversa

XML Treatment for Neocarpia rhizophorae
